# Prognostic value of GRACE and CHA2DS2-VASc score among patients with atrial fibrillation undergoing percutaneous coronary intervention

**DOI:** 10.1080/07853890.2021.2004321

**Published:** 2021-11-18

**Authors:** Tingting Guo, Ziwei Xi, Hong Qiu, Yong Wang, Jianfeng Zheng, Kefei Dou, Bo Xu, Shubin Qiao, Weixian Yang, Runlin Gao

**Affiliations:** a Thrombosis Center, State Key Laboratory of Cardiovascular Disease, Fuwai Hospital, National Center for Cardiovascular Diseases, Peking Union Medical College, Chinese Academy of Medical Sciences, Beijing, China}; b Department of Cardiology, Coronary Artery Disease Center, State Key Laboratory of Cardiovascular Disease, Fuwai Hospital, National Center for Cardiovascular Diseases, Peking Union Medical College, Chinese Academy of Medical Sciences, Beijing, China

**Keywords:** GRACE, CHA2DS2-VASc score, risk score, atrial fibrillation, percutaneous coronary intervention

## Abstract

**Aims:**

The GRACE and CHA2DS2-VASc risk score are developed for risk stratification in patients with acute coronary syndrome and AF, respectively. We aimed to assess the predictive performance of the GRACE score and CHA2DS2-VASc score among patients with atrial fibrillation (AF) undergoing percutaneous coronary intervention (PCI).

**Methods:**

Consecutive patients with a diagnosis of AF admitted to our hospital for PCI between January 2016 and December 2018 were included and followed up for at least 1 year. The primary endpoint was a composite of major adverse cardiac events (MACEs) including all-cause mortality, repeat revascularization, myocardial infarction, or ischaemic stroke.

**Results:**

A total of 1452 patients were identified. Cox regression demonstrated that the GRACE (HR 1.014, 95% CI 1.008–1.020, *p* < 0.001) but not the CHA2DS2-VASc score was associated with the risk of MACEs. Both GRACE and CHA2DS2-VASc scores were predictive of all-cause mortality with HR of 1.028 (95% CI 1.020–1.037, *p* < 0.001) and 1.334 (95% CI 1.107–1.632, *p* = 0.003). Receiver operating characteristic analyses showed both scores had similar discrimination capacity for all-cause mortality (C-statistic: 0.708 for GRACE vs. 0.661 for CHA2DS2-VASc, *p* = 0.299). High GRACE score was also significantly associated with increased risk of ischaemic stroke (HR 1.018, 95% CI 1.005–1.031, *p* = 0.006) and major bleeding (HR 1.012, 95% CI 1.001–1.024, *p* = 0.039), whereas high CHA2DS2-VASc score was not.

**Conclusions:**

High GRACE score but not CHA2DS2-VASc score were both associated with an increased risk of MACEs after PCI in patients with AF. The GRACE and CHA2DS2-VASc scores have similar predictive performance for predicting all-cause mortality.

**Key messages:**
- In patients with AF undergoing PCI, increasing GRACE but not CHA2DS2-VASc scores was independently associated high risk of MACEs.
- The GRACE score could also help identify patients at higher risk of stroke and major bleeding.
- Both GRACE and CHA2DS2-VASc scores showed good ability in the prediction of all-cause mortality.

## Introduction

Atrial fibrillation (AF) represents the most common cardiac arrhythmia and confers a substantial risk of mortality and stroke. Approximately 20–40% of patients with AF have co-existing coronary artery disease (CAD), a sizeable proportion of whom will require percutaneous coronary intervention (PCI) [1]. The optimal management strategy for patients with AF undergoing PCI remains a challenge in clinical practice [2]. Current clinical guidelines recommend individualised risk assessment in AF patients and CAD patients to predict ischaemic risk and guide therapeutic interventions [3,4].

The Global Registry of Acute Coronary Events (GRACE) risk score was developed for risk stratification in patients with acute coronary syndrome and has been incorporated into clinical guidelines [4–7]. The use of the GRACE score to identify high-risk patients plays an important role in the management of CAD and is recommended in patients with suspected ACS across all international guidelines. The GRACE score, which includes clinical variables, the electrocardiogram, and cardiac biomarkers, has become the gold standard score and has been widely accepted. Moreover, in a previous study, the GRACE score showed excellent diagnostic performance for the prediction of new-onset AF in patients with acute myocardial infarction [8].

The CHA2DS2-VASc score is an effective tool for the assessment of thromboembolic risk and guiding antithrombotic therapy in patients with AF [3]. The clinical utility of CHA2DS2-VASc score has been well validated in various clinical settings [9–11]. Several studies have demonstrated that a higher CHA2DS2-VASc score was independently associated with increased risk of mortality and adverse outcomes in different groups of patients with CAD regardless of the presence of AF [12,13]. In addition, some studies indicate that the risk score developed for AF has an even greater prognostic value in patients with ACS who do not have AF [14].

The number of patients with AF undergoing PCI is increasing and has become an emerging problem with the ever-growing elderly population. About 5–10% of patients referred to coronary angiography present with AF [15]. However, there is still no dedicated scoring system for risk stratification in patients with AF undergoing PCI. The aims of the present study are to assess the performance of the GRACE score and CHA2DS2-VASc score for the prediction of adverse outcomes among patients with AF undergoing PCI and compare them.

## Methods

### Study population

This retrospective observational study included a total of 1452 consecutive patients admitted to our institution for PCI with a diagnosis of AF between January 1, 2016 and December 31, 2018. Patients with previous, persistent, permanent, or paroxysmal atrial fibrillation diagnosed before or during the hospitalisation were included if they aged ≥18 years and underwent urgent or elective PCI with at least one drug-eluting stent. Patients who had missing data of individual components of risk scores or underwent unsuccessful PCI were excluded. Patient demographic information, medical history, laboratory assessments, medication, angiographic data, and procedural data were collected retrospectively from medical records. Patients were followed up by telephone interviewers using standardised questionnaires at 6 and 12 months after the index procedure. The follow-up is part of the routine treatment for patients undergoing interventional therapy in the cardiac catheterisation laboratory of our centre.

This study used data from an institutional registry approved by the Ethics Committee of Fuwai Hospital (ChiCTR2100047090). And the committee approved that the study protocol and inform consent was not required for the de-identified data and the non-interventional observational nature of our study. The study complied with the Declaration of Helsinki [16].

### Risk scores

The GRACE and CHA2DS2-VASc scores were calculated for each individual patient. Patients were classified into 3 categories according to the GRACE score based on age, heart rate, systolic blood pressure, Killip class, creatinine and cardiac enzyme levels, ST-segment deviation in the electrocardiogram, and cardiac arrest at admission (low-risk: 108 points, intermediate-risk: 109–140 points, and high-risk: >140 points) [17]. Patients were also divided into 3 groups according to the CHA2DS2-VASc score which included congestive heart failure, hypertension, age, diabetes, vascular disease, and sex (low-risk: 0–1 point, intermediate-risk: 2–3 points, and high-risk: ≥4 points) [3].

### Endpoints

A composite of MACEs including all-cause mortality, repeat revascularization, myocardial infarction (MI), or ischaemic stroke was defined as the primary endpoint. The secondary endpoints were individual all-cause mortality and ischaemic stroke. MI was defined according to the Third Universal Definition of Myocardial Infarction [18]. Stroke was identified using physician-reported diagnosis. The safety endpoints included minor bleeding and major bleeding events. Any bleeding event that required non-surgical medical intervention by a healthcare professional would be identified as a minor bleeding event. The major criteria of major bleeding were as the following: overt bleeding plus a haemoglobin decrease ≥ 3 g/dL, any transfusion with overt bleeding, cardiac tamponade, bleeding requiring surgical intervention for control, bleeding requiring intravenous vasoactive agents, intracranial haemorrhage, intraocular bleeding that compromised vision, or fatal bleeding [19].

### Statistical analysis

We analysed and compared the baseline characteristics including demographics, medical history, angiographic features, procedural characteristics between the patients who had adverse events and those who did not. Categorical variables were expressed as numbers (frequencies) and continuous variables as mean ± standard deviation or median and interquartile range as appropriate. Continuous variables were compared using the independent Student’s *t*-tests or Mann-Whitney test and categorical variables using the Chi-square test or Fisher’s exact test in accordance with the distribution. Cox proportional hazard models were used to calculate the hazard ratio (HR) and 95% confidence intervals (CI) to assess the independent contribution of the GRACE and CHA2DS2-VASc score to the composite primary endpoint. Variables included in the GRACE or CHA2DS2-VASc score were not entered into the model. Multivariable Cox proportional hazard models were constructed by other variables that were associated with events of interest in previous studies and obtained *P* values < 0.1 in the univariate analysis.

Receiver operating characteristic (ROC) curve analysis was performed to assess the discrimination performance of the two risk scores used as continuous variables in the prediction of all-cause mortality and ischaemic stroke. The statistical significance of the difference between two areas under the receiver operating curve (AUCs) was tested using the DeLong method. We used Hosmer-Lemeshow goodness-of-fit test to assess the calibration of risk scores. We also calculated the net classification improvement (NRI), which represents the average weighted improvement in discrimination, and integrated discrimination improvement (IDI) index as described in previous studies to compare the two risk scores [20,21].

All reported *p* values were two-sided, and *p* < 0.05 was considered to indicate statistical significance for all analyses. Statistical analyses were performed using STATA software version 15.0 (STATA Corp LP, College Station, TX, USA).

## Results

### Patient characteristics

**Figure 1.**
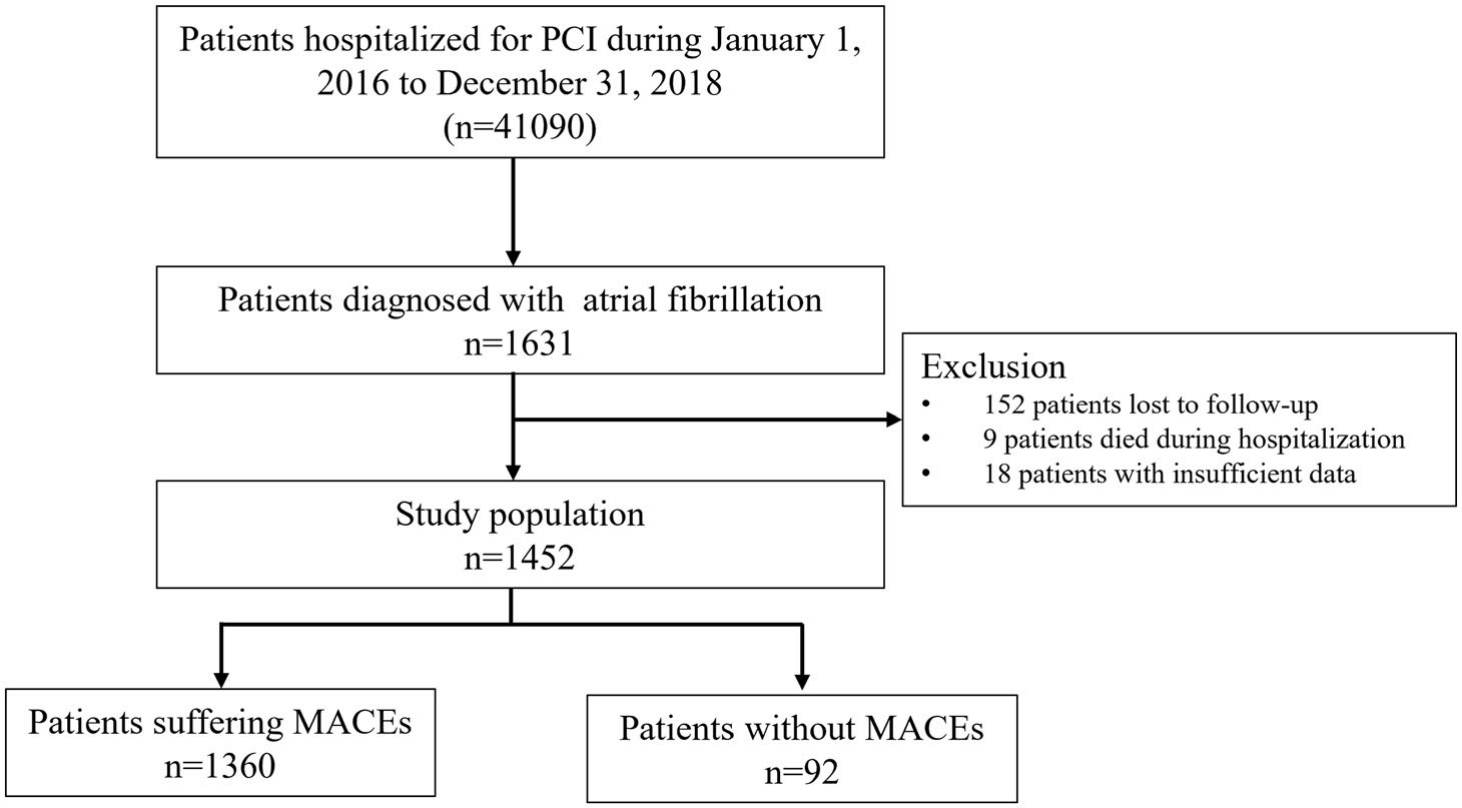
Flow chart illustrating cohort selection for the main analysis. MACEs: major adverse cardiac events, PCI: percutaneous coronary intervention.

The study population included 1452 PCI patients with a history of or newly diagnosis AF. The selection of the study populations is summarised in Figure 1. The average age of the total population was 66.4 ± 9.3 years (range 29.2–92.0 years) and 24.8% (*n* = 362) were female. A total of 226 (15.6%) patients underwent urgent procedures. Based on the GRACE score, 268 patients (16.8%) were at low risk, 650 patients (44.8%) were at intermediate risk, and 534 patients (36.8%) were at high risk. Regarding the CHA2DS2-VASc score, 312 patients (21.5%) presented with a score between 0 and 1, 617 (42.5%) had a score between 2 and 3 and 523 (36.0%) had a score above 3.

MACEs occurred in 92 patients (6.3%) and all-cause mortality occurred in 27 patients (1.9%) during follow-up of 12 months. Baseline demographic and clinical characteristics were compared between patients who had MACEs and all-cause mortality and patients who did not, as presented in Table 1. Patients who had MACEs were older and had higher body mass index. They were also more likely to have the three-vessel disease. In patients suffering death, advanced age and female were more frequent. Renal and liver insufficiency were also more common in patients who died during follow-up. The proportions of patients diagnosed with STEMI (12.1% vs. 22.8%) and NSTEMI (9.5% vs. 19.6%) were considerably higher in patients suffering MACEs.

**Table 1.** Patient characteristics of the study population.

Variable	All patients (*n* = 1452)	MACEs			All-cause mortality		
		No (*n* = 1360)	Yes (*n* = 92)	*P* value	No (*n* = 1425)	Yes (*n* = 27)	*P* value
Age (years)	66.4 ± 9.3	66.2 ± 9.2	69.0 ± 10.6	0.005	66.3 ± 9.2	73.9 ± 13.4	0.007
Age > 75y	293 (20.2%)	270 (19.9%)	23 (25.0%)	0.234	281 (19.7%)	12 (44.4%)	0.002
Female participant	362 (24.9%)	335 (24.6%)	27 (29.3%)	0.312	350 (24.6%)	12 (44.4%)	0.018
Systolic blood pressure (mmHg)	131.9 ± 17.0	131.8 ± 16.8	132.4 ± 18.4	0.746	131.9 ± 16.8	132.4 ± 20.7	0.859
Heart rate	77.0 ± 20.0	76.8 ± 18.7	79.6 ± 22.3	0.168	76.9 ± 19.0	79.4 ± 20.4	0.521
Body mass index (kg/m^2^)	25.8 (23.8, 27.8)	25.8 (23.8, 27.9)	25.0 (23.0, 27.4)	0.030	25.8 (23.8, 27.9)	24.2 (22.5, 25.6)	0.013
Ever-smoking	814 (56.1%)	764 (56.2%)	50 (54.3%)	0.732	802 (56.3%)	12 (44.4%)	0.220
Alcohol abuse	115 (7.9%)	108 (7.9%)	7 (7.6%)	0.909	110 (7.7%)	5 (18.5%)	0.040
Presentation of congestive heart failure	118 (8.1%)	104 (7.6%)	14 (15.2%)	0.010	112 (7.9%)	6 (22.2%)	0.007
Hypertension	1098 (75.6%)	1029 (75.7%)	69 (75.0%)	0.886	1076 (75.5%)	22 (81.5%)	0.474
Hyperlipidemia	1284 (88.4%)	1205 (88.5%)	80 (87.0%)	0.648	1261 (88.5%)	23 (85.2%)	0.595
Diabetes mellitus	566 (39.0%)	534 (39.3%)	32 (34.8%)	0.394	555 (38.9%)	11 (40.7%)	0.850
Stroke	326 (22.5%)	307 (22.6%)	19 (20.7%)	0.669	320 (22.5%)	6 (22.2%)	0.977
Peripheral artery disease	244 (16.8%)	227 (16.7%)	17 (18.5%)	0.657	242 (17.0%)	2 (7.4%)	0.187
Renal insufficiency	101 (7.0%)	90 (6.6%)	11 (12.0%)	0.051	92 (6.5%)	9 (33.3%)	<0.001
Diagnosis at admission for CAD							
Stable CAD	319 (22.0%)	303 (22.3%)	16 (17.6%)	<0.001	317 (22.2%)	2 (7.4%)	<0.001
Unstable angina	800 (55.1%)	763 (56.1%)	37 (40.2%)		791 (55.5%)	9 (33.3%)	
NSTEMI	147 (10.1%)	129 (9.5%)	18 (19.6%)		140 (9.8%)	7 (25.9%)	
STEMI	186 (12.8%)	165 (12.1%)	21 (22.8%)		177 (12.4%)	9 (33.3%)	
Prior PCI	300 (20.7%)	279 (20.5%)	21 (22.8%)	0.586	295(20.7%)	5 (18.5%)	0.781
Prior CABG	34 (2.3%)	31 (2.3%)	3 (3.3%)	0.547	34 (2.4%)	0 (0.0%)	0.417
Prior MI	314 (21.6%)	287 (21.1%)	27 (29.3%)	0.063	307 (21.5%)	7 (25.9%)	0.584
Major bleeding history	45 (3.1%)	40 (2.9%)	5 (5.4%)	0.182	41 (2.9%)	4 (14.8%)	<0.001
LVEF	57.8 ± 9.0	58.0 ± 9.0	55.8 ± 8.9	0.057	57.9 ± 9.0	53.2 ± 9.6	0.008
LAAP	40.8 ± 13.1	40.7 ± 13.5	41.1 ± 6.3	0.808	40.7 ± 13.3	42.0 ± 6.6	0.266
LVEDD	50.2 ± 6.0	50.2 ± 5.9	50.3 ± 6.6	0.905	50.2 ± 6.0	50.1 ± 6.3	0.908
Three-vessel disease	570 (39.3%)	517 (38.0%)	53 (57.6%)	<0.001	557 (39.1%)	13 (48.1%)	0.340
Left main disease	168 (11.6%)	153 (11.2%)	16 (17.4%)	0.071	164 (11.5%)	4 (14.8%)	0.595
CHA2DS2-VASc	3 (2, 4)	3 (2, 4)	3 (2, 4.75)	0.055	3 (2, 4)	4 (3, 5)	0.002
Low (0, 1)	312 (21.5%)	309 (21.7%)	3 (11.1%)	0.089	309 (21.7%)	3 (11.1%)	0.089
Intermediate (2, 3)	617 (42.5%)	608 (42.7%)	9 (33.3%)		608 (42.7%)	9 (33.3%)	
High (≥4）	523 (36.0%)	508 (35.6%)	15 (55.6%)		508 (35.6%)	15 (55.6%)	
GRACE risk score	131 (115, 151)	131 (114, 150)	143 (122, 167)	<0.001	131 (115, 151)	168 (124, 220)	<0.001
Low (≤108)	268 (18.6%)	256 (18.6%)	3 (11.1%)	0.005	265 (18.6%)	3 (11.1%)	0.005
Intermediate (109–140)	650 (44.8%)	644 (45.2%)	6 (22.2)		644 (45.2%)	6 (22.2%)	
High (>140)	534 (36.8%)	516 (36.2%)	18 (66.7%)		516 (36.2%)	18 (66.7%)	

Renal insufficiency was defined as eGFR< 60 ml/min at admission or having a history of chronic kidney disease stage 3 to 5. Alcohol abuse was defined as consuming >60 g of alcohol a day for >5 years. MACEs: major adverse cardiac events, CAD: coronary artery disease, PCI: percutaneous coronary intervention, MI: myocardial infarction, CABG: coronary artery bypass grafting, LVEF: left ventricular ejection fraction, LAAP: left atrium diameter, LVEDD: left ventricular end-diastolic dimension.

### Clinical outcomes

Patients who met the primary endpoint had significantly higher GRACE scores than patients who did not, while the CHA2DS2-VASc scores were similar between them as a continuous score and as a categorical score (Table 1). Both the GRACE and CHA2DS2-VASc risk scores used as continuous scores were associated with all-cause mortality after PCI in patients with AF, while the GRACE score used as a categorical score could also predict all-cause mortality.

**Figure 2.**
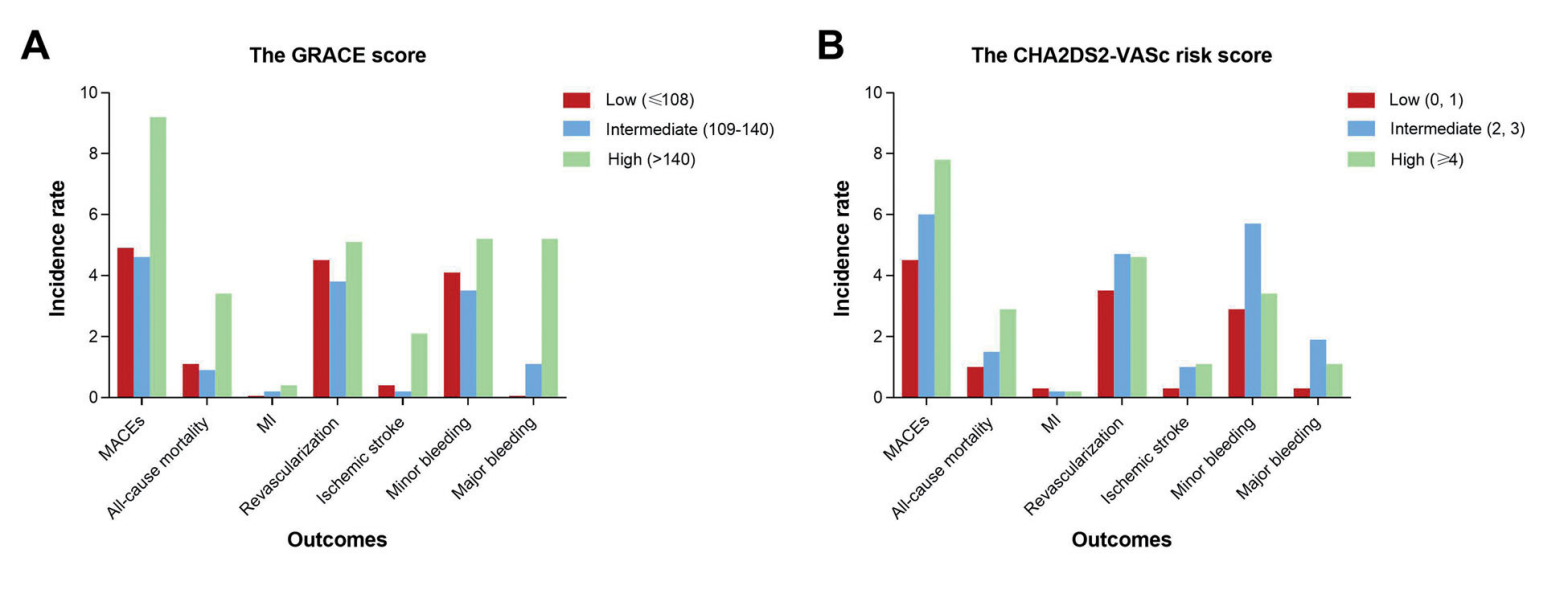
Incidence rates of adverse outcomes at 1 year in the GRACE and CHA2DS2-VASc risk categories. The incidences of endpoints including major adverse cardiac events, all-cause death, myocardial infarction, revascularization were compared, ischaemic stroke, and bleeding events between patients stratified according to the GRACE and CHA2DS2-VASc risk categories. MACEs: major adverse cardiac events, MI: myocardial infarction.

The incidences of adverse outcomes in the GRACE and CHA2DS2-VASc risk categories were presented in Table 2 and Figure 2. The composite primary endpoint occurred in 92 (6.3%) patients of the study cohort. The incidence rates of all-cause mortality, myocardial infarction, repeat revascularization, and stroke were 1.9%, 0.2%, 4.4%, and 0.9% respectively. A total of 19 (1.3%) patients had major bleeding events during follow-up. The multivariate Cox proportional hazards model revealed that higher GRACE score was independently associated with increased risk of MACEs, no matter that the GRACE score was used as a continuous (HR 1.014, 95% CI 1.008–1.020, *p* < 0.001) or categorical (HR 1.561, 95% CI 1.150–2.118, *p* = 0.004) score. However, a high CHA2DS2-VASc score was not a predictor of MACEs after PCI in multivariate analysis. Furthermore, the GRACE score was a strong and independent predictor of all-cause mortality (continuous: HR 1.028, 95% CI 1.020–1.037, *p* < 0.001, categorical: HR 2.315, 95% CI 1.238–4.326, *p* = 0.009) as well as ischaemic stroke (continuous: HR 1.018, 95% CI 1.005–1.031, *p* = 0.006, categorical: HR 4.997, 95% CI 1.496–15.965, *p* = 0.009). Patients with a high CHA2DS2-VASc score had significantly higher risk of all-cause mortality (continuous: HR 1.334, 95% CI 1.107–1.632, *p* = 0.003, categorical: HR 1.819, 95% CI 1.034–3.201, *p* = 0.038) but not ischaemic stroke. The results of the multivariate Cox proportional hazards models were presented in the Supplementary Material.

**Table 2.** Clinical outcomes at 1 year in the GRACE and CHA2DS2-VASc risk categories.

Outcomes	All patients (*n* = 1452)	GRACE			CHA2DS2-VASc		
		Low (≤108) (*n* = 268)	Intermediate (109–140) (*n* = 650)	High (>140) (*n* = 534)	Low (0, 1) (*n* = 312)	Intermediate (2, 3) (*n* = 617)	High (≥4) (*n* = 523)
MACEs	92 (6.3%)	13 (4.9%)	30 (4.6%)	49 (9.2%)	14 (4.5%)	37 (6.0%)	41 (7.8%)
All-cause mortality	27 (1.9%)	3 (1.1%)	6 (0.9%)	18 (3.4%)	3 (1.0%)	9 (1.5%)	15 (2.9%)
Myocardial infarction	3 (0.2%)	0 (0.0%)	1 (0.2%)	2 (0.4%)	1 (0.3%)	1 (0.2%)	1 (0.2%)
Repeat revascularization	64 (4.4%)	12 (4.5%)	25 (3.8%)	27 (5.1%)	11 (3.5%)	29 (4.7%)	24 (4.6%)
Ischaemic stroke	13 (0.9%)	1 (0.4%)	1 (0.2%)	11 (2.1%)	1 (0.3%)	6 (1.0%)	6 (1.1%)
Minor bleeding	62 (4.3%)	11 (4.1%)	23 (3.5%)	28 (5.2%)	9 (2.9%)	35 (5.7%)	18 (3.4%)
Major bleeding	19 (1.3%)	0 (0.0%)	7 (1.1%)	12 (2.2%)	1 (0.3%)	12 (1.9%)	6 (1.1%)

MACEs: major adverse cardiac events.

Regarding the safety endpoints, neither the GRACE or CHA2DS2-VASc score was associated with an increased risk of minor bleeding events. The GRACE score was predictive of major bleeding events (continuous: HR 1.012, 95% CI 1.001–1.024, *p* = 0.039, categorical: HR 2.880, 95% CI 1.291–6.442, *p* = 0.010) while the CHA2DS2-VASc score was not.

### Performance of risk scores for predicting outcomes

**Figure 3.**
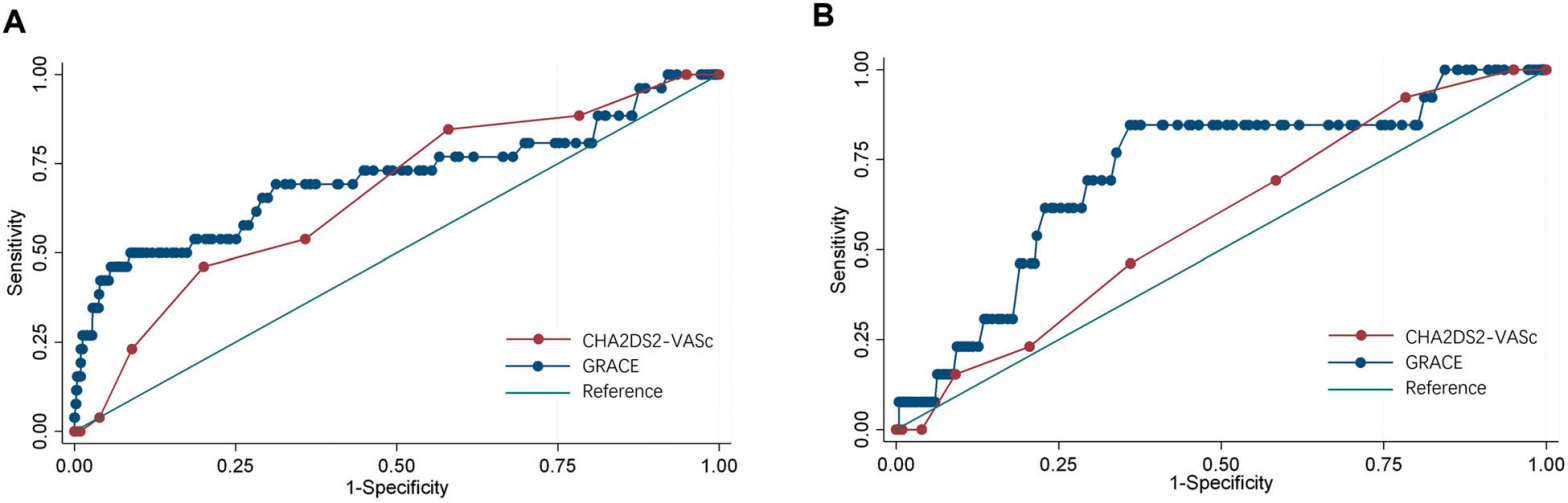
ROC curves of the GRACE and CHA2DS2-VASc risk scores for predicting (A) all-cause mortality, and (B) ischaemic stroke. ROC curves of the GRACE and CHA2DS2-VASc risk scores to predict the incidence of all-cause mortality, and ischaemic stroke.

Figure 3 presented the ROC curves of the GRACE or CHA2DS2-VASc scores for all-cause mortality and ischaemic stroke. For prediction of all-cause mortality, ROC analyses showed that both GRACE or CHA2DS2-VASc scores were able to discriminate all-cause mortality (GRACE: AUC 0.708, 95% CI 0.579–0.837, CHA2DS2-VASc: AUC 0.661, 95% CI 0.557–0.765). And the GRACE score had comparable discrimination capacity compared to the and CHA2DS2-VASc score (*p* = 0.299). Calibration was acceptable for both risk scores (H-L *p* value: 0.494 for GRACE 0.231 for CHA2DS2-VASc). The NRI analysis showed that the GRACE score was more accurately associated with all-cause mortality compared with the CHA2DS2-VASc score (NRI: 8.2%). Moreover, the IDI also indicated that the GRACE score had better discrimination (IDI 1.64%, 95% CI 1.41%-1.87%, *p* < 0.001). Furthermore, the GRACE score had considerable discrimination accuracy for ischaemic stroke with an AUC of 0.715 (95% CI 0.574–0.856) while the CHA2DS2-VASc score has relatively poor diagnostic performance with an AUC of 0.580 (95% CI 0.439–0.721).

## Discussion

Our study assessed the performance of the GRACE score and CHA2DS2-VASc scores in predicting adverse outcomes among patients with AF undergoing PCI. The results demonstrated that high GRACE but not a high CHA2DS2-VASc score was independently associated with an increased risk of MACEs. For the prediction of all-cause mortality, the GRACE and CHA2DS2-VASc scores respectively provided good discrimination, and the prognostic performance of the above two risk scores was comparable. In addition, the GRACE score also had an acceptable value in the prediction of ischaemic stroke and could provide prognostic information for major bleeding, while the CHA2DS2-VASc score could not.

Appropriate management based on early individualised risk stratification for each patient is recommended by current guidelines, aiming to identify high-risk patients who require intensive care and reduce unnecessary treatments for low-risk patients. There have been lots of established risk scores for patients with CAD. However, none of them is specifically developed for patients with AF undergoing PCI. Of note, the coexistence of AF and the need for PCI lead to an increased risk of thrombotic and bleeding events. Previous studies have shown that AF is common in the setting of CAD and is associated with a significantly higher risk of adverse cardiovascular outcomes including mortality, reinfarction, and ischaemic stroke [22]. Substantial increases in the risk of poor outcomes suggest AF can no longer be considered a non-severe event in patients with CAD. The fact that cardiac adverse events and AF share similar pathophysiological pathways supports the increased risk of poor outcomes following PCI in AF patients [23]. It is reported that AF has multiple adverse hemodynamic effects on patients, such as loss of atrial contraction, rapid ventricular rates, loss of atrioventricular synchrony, and an irregular RR interval, leading to a decrease in cardiac output [24]. At the same time, AF might be a marker for illness, inflammation, and structural heart disease in the elderly [25–27].

The GRACE score is derived from a large multinational registry of patients admitted for the entire spectrum of ACS [5]. The GRACE score is widely applicable to patients in various hospital settings to improve prognostication and promote consistency in management. Previous studies have validated the accuracy of the GRACE score in contemporary cohorts of patients across a wide range of ACS. Numerous studies have demonstrated that despite significant temporal improvement in treatment and outcomes of ACS patients over the last two decades, the GRACE score remained an effective tool for the prediction of all-cause mortality rates in these patients. Besides ACS patients, the GRACE score also has appropriate predictive power and good calibration and clinical applicability in other populations such as patients with diabetes [28], Takotsubo syndrome [29], or pulmonary embolism [20]. Several components included by the GRACE score have also been proved to be risk factors of an adverse prognosis in patients with AF [3], which partially explain the good performance of the GRACE risk score in our study. Although the GRACE score was not designed to identify the risk of stroke, the increased GRACE score was shown to be strongly associated with a higher risk of stroke in our study, which was in accordance with findings from Álvarez-Álvarez et al. [30] Moreover, Álvarez-Álvarez and colleagues found that discriminative ability of GRACE risk score to predict ischaemic stroke was similar to CHA2DS2VASc, even in patients with AF. Given the association between high GRACE score and increased risk of ischaemic events, our results supported the potential application of the GRACE risk score in patients with AF after PCI to identifying high-risk patients who might benefit from aggressive antithrombotic treatment strategies.

A growing body of evidence has been built about the use of clinical risk scores to predict adverse outcomes, particularly ischaemic events and major bleeding events. Bleeding complications have aroused increased concern over the last decades due to the wide application of invasive strategies and antithrombotic treatments, especially in patients with AF undergoing PCI who require treatment with the combination of oral anticoagulation and antiplatelet therapy [2]. Our results suggested that the GRACE score could help to identify patients at high risk of bleeding, which was in accordance with previous studies. It has been proposed that the GRACE score could predict in-hospital major bleeding with a similar or even better predictive accuracy of the American College of Cardiology/American Heart Association guidelines (CRUSADE) risk score [31].

The CHA2DS2-VASc score has been widely used for ischaemic stroke risk stratification in patients with AF [32] as well as patients with CAD and treated with coronary stent implantation [33]. Moreover, in patients without AF, increasing CHA2DS2-VASc score remains associated with an increased risk of MACEs [10,13,34]. A network meta-regression has shown that the CHA2DS2-VASc score was associated with an increased risk of all-cause mortality, both as continuous and as categorical scores [35]. The CHA2DS2-VASc score is comprised of several traditional risk factors that contribute to the poor prognosis of cardiovascular disease. Advanced age, hypertension, stroke/transient ischaemic attack, diabetes, and congestive heart failure were all reported to be associated with adverse outcomes in patients with CAD [7]. Hence, the utility of the CHA2DS2-VASc score in patients with CAD is theoretically reasonable. In addition, the CHA2DS2-VASc score provides physicians a simple, fast, and comprehensive way for risk estimation which can be easily assessed bedside and requires no calculators or computers.

The CHA2DS2-VASc score showed marginally better ability in predicting the appearance of MCCEs over the GRACE score in patients with angioplasty in a study from Trantalis et al. [36] On the contrary, the CHA2DS2-VASc score only achieved suboptimal discrimination for ischaemic stroke with an AUC of 0.580 which indicate modest discriminatory power and poor specificity in our study, while the GRACE was shown to be relatively predictive for stroke after PCI with an AUC of 0.715. The renal function parameter was included as an element in the GRACE score but not in the CHA2DS2-VASc score, which might be an explanation of the better performance of the former. A study from Piccini et al. found that a modified CHADS2 score by adding 2 points for creatinine clearance <60 mL/min improved net stroke risk reclassification over the CHADS2 and CHA2DS2-VASc score [37]. Interestingly, a study from Shuvy et al. suggested that the addition of the CHA2DS2-VASc score to the GRACE score in ACS patients could significantly improve risk stratification of patients with low and intermediate risk [38]. In terms of bleeding, our findings are in line with a previous study that validated the CHA2DS2-VASc score in non-AF patients undergoing PCI from Capodanno et al. and suggested that the CHA2DS2-VASc score had modest discrimination for major bleeding [39]. However, the ENTRUST-AF PCI subgroup analysis from Goette et al. demonstrated that a high CHA2DS2-VASc score was a marker for occurrence of major bleeding [33].

Although we did not report information on antithrombotic therapy in the present study due to lack of related data, it should be noted that antithrombotic treatment is of great importance for patients with AF or with stent implantation, whereas the optimal antithrombotic treatment regimen for AF patients undergoing PCI remains challenging in clinical practice. An individual assessment using risk scores is reported to be necessary for the decision-making process of selecting antithrombotic agents in this population, particularly the CHA2DS2-VASc score which could clearly determine whether or not a patient should receive oral anticoagulant therapy.

There are some limitations in our study that warrants attention. First, the present study is a single-centre retrospective study there may be residual confounding bias inherent in the observational study design. Our findings need to be further validated in the contemporary large cohorts before being extended to all patients. Nonetheless, the sample size of our study which included 1452 patients with AF undergoing PCI is relatively larger than most previous cohort studies on this population. Second, as mentioned above, the antithrombotic treatment regimen plays an important role in the management of patients with AF undergoing PCI, but it is not recorded in our study. Third, we did not analyse the difference in the performance of the two risk scores between different subtypes of AF due to a lack of sufficient data. However, a previous study has observed that there was no major difference in outcome between AF subtypes in patients with AF after myocardial infarction [22]. Fourth, the incidences of adverse clinical outcomes were relatively low, which could be partly explained by underreporting in the follow-up. Thus, our findings needed to be interpreted with caution. Additionally, the fact that most of the clinical outcomes investigated in our study were competitive should be taken into consideration when interpreting our findings.

## Conclusions

In patients with AF undergoing PCI, increasing GRACE but not CHA2DS2-VASc scores were independently associated high risk of MACEs. Both the GRACE and CHA2DS2-VASc scores demonstrated good discriminatory ability in predicting all-cause mortality and they have comparable performance for prediction. The GRACE score can also help identify patients at high risk of ischaemic stroke and major bleeding, while the CHA2DS2-VASc score cannot. Our findings provide evidence for the application of the GRACE and CHA2DS2-VASc scores in contemporary patients with AF undergoing PCI and support the preferential use of the GRACE score. A more accurate and exclusive risk stratification tool is needed to help clinical decision-making in this population.

## Supplementary Material

Supplemental Material

## Data Availability

The deidentified participant data will be shared on a request basis. Please directly contact the corresponding author to request data sharing.
