# Supplementary material for: Prognostic value of GRACE and CHA2DS2-VASc score among patients with atrial fibrillation undergoing percutaneous coronary intervention
Source: Ann Med. 2021 Nov 18;53(1):2217–26. doi: 10.1080/07853890.2021.2004321 (PMC8604500; doi:10.1080/07853890.2021.2004321)
Supplement: Supplemental Material [file IANN_A_2004321_SM8795.docx]

Supplementary Table I Results of multivariable Cox proportional hazard models included the GRACE score as a continuous variable for MACEs.

| **Variables** | **HR** | **95% CI** | | **p Value** |
| --- | --- | --- | --- | --- |
|  |  | **Lower** | **Upper** |  |
| The GRACE score | 1.014 | 1.008 | 1.020 | <0.001 |
| Female | 1.252 | 0.732 | 2.144 | 0.412 |
| Body mass index | 0.955 | 0.897 | 1.016 | 0.146 |
| Ever-smoking | 1.100 | 0.671 | 1.803 | 0.706 |
| Hypertension | 0.994 | 0.605 | 1.634 | 0.982 |
| Hyperlipidemia | 0.817 | 0.442 | 1.511 | 0.520 |
| Diabetes mellitus | 0.846 | 0.544 | 1.313 | 0.455 |
| Stroke history | 0.821 | 0.490 | 1.377 | 0.455 |
| Prior PCI | 1.157 | 0.692 | 1.934 | 0.579 |
| Prior MI | 1.395 | 0.864 | 2.254 | 0.174 |
| Major bleeding history | 1.665 | 0.664 | 4.176 | 0.277 |

Supplementary Table II Results of multivariable Cox proportional hazard models included the GRACE score as a categorical variable for MACEs.

| **Variables** | **HR** | **95% CI** | | **p Value** |
| --- | --- | --- | --- | --- |
|  |  | **Lower** | **Upper** |  |
| The GRACE score | 1.561 | 1.150 | 2.118 | 0.004 |
| Female | 1.257 | 0.735 | 2.150 | 0.404 |
| Body mass index | 0.943 | 0.886 | 1.003 | 0.063 |
| Ever-smoking | 1.035 | 0.634 | 1.691 | 0.891 |
| Hypertension | 1.034 | 0.631 | 1.694 | 0.893 |
| Hyperlipidemia | 0.871 | 0.472 | 1.608 | 0.659 |
| Diabetes mellitus | 0.856 | 0.554 | 1.324 | 0.485 |
| Stroke history | 0.822 | 0.490 | 1.377 | 0.456 |
| Prior PCI | 1.053 | 0.631 | 1.759 | 0.842 |
| Prior MI | 1.438 | 0.886 | 2.332 | 0.141 |
| Major bleeding history | 1.787 | 0.716 | 4.460 | 0.214 |

Supplementary Table III Results of multivariable Cox proportional hazard models included the GRACE score as a continuous variable for all-cause mortality.

| **Variables** | **HR** | **95% CI** | | **p Value** |
| --- | --- | --- | --- | --- |
|  |  | **Lower** | **Upper** |  |
| The GRACE score | 1.028 | 1.020 | 1.037 | <0.001 |
| Female | 2.673 | 1.029 | 6.943 | 0.043 |
| Body mass index | 0.932 | 0.833 | 1.043 | 0.221 |
| Ever-smoking | 1.510 | 0.582 | 3.915 | 0.397 |
| Hypertension | 1.357 | 0.478 | 3.854 | 0.567 |
| Hyperlipidemia | 0.527 | 0.176 | 1.583 | 0.254 |
| Diabetes mellitus | 1.153 | 0.512 | 2.599 | 0.731 |
| Stroke history | 0.679 | 0.258 | 1.784 | 0.432 |
| Prior PCI | 1.130 | 0.407 | 3.139 | 0.815 |
| Prior MI | 1.052 | 0.424 | 2.607 | 0.913 |
| Major bleeding history | 4.724 | 1.468 | 15.206 | 0.009 |

Supplementary Table IV Results of multivariable Cox proportional hazard models included the GRACE score as a categorical variable for all-cause mortality.

| **Variables** | **HR** | **95% CI** | | **p Value** |
| --- | --- | --- | --- | --- |
|  |  | **Lower** | **Upper** |  |
| The GRACE score | 2.315 | 1.238 | 4.326 | 0.009 |
| Female | 2.181 | 0.838 | 5.677 | 0.110 |
| Body mass index | 0.899 | 0.803 | 1.007 | 0.066 |
| Ever-smoking | 1.094 | 0.426 | 2.813 | 0.852 |
| Hypertension | 1.528 | 0.554 | 4.219 | 0.413 |
| Hyperlipidemia | 0.741 | 0.253 | 2.170 | 0.585 |
| Diabetes mellitus | 1.103 | 0.506 | 2.407 | 0.805 |
| Stroke history | 0.670 | 0.259 | 1.729 | 0.407 |
| Prior PCI | 0.862 | 0.315 | 2.363 | 0.773 |
| Prior MI | 1.097 | 0.441 | 2.730 | 0.843 |
| Major bleeding history | 5.464 | 1.796 | 16.624 | 0.003 |

Supplementary Table V Results of multivariable Cox proportional hazard models included the GRACE score as a continuous variable for stroke.

| **Variables** | **HR** | **95% CI** | | **p Value** |
| --- | --- | --- | --- | --- |
|  |  | **Lower** | **Upper** |  |
| The GRACE score | 1.018 | 1.005 | 1.031 | 0.006 |
| Female | 0.311 | 0.055 | 1.752 | 0.185 |
| Body mass index | 0.909 | 0.748 | 1.105 | 0.340 |
| Ever-smoking | 0.596 | 0.162 | 2.201 | 0.438 |
| Hypertension | 0.921 | 0.226 | 3.751 | 0.908 |
| Hyperlipidemia | 0.509 | 0.103 | 2.509 | 0.406 |
| Diabetes mellitus | 0.336 | 0.072 | 1.562 | 0.164 |
| Stroke history | 1.901 | 0.534 | 6.763 | 0.321 |
| Prior PCI | 4.949 | 1.342 | 18.256 | 0.016 |
| Prior MI | 0.845 | 0.206 | 3.471 | 0.815 |
| Major bleeding history | 3.278 | 0.611 | 17.577 | 0.166 |

Supplementary Table VI Results of multivariable Cox proportional hazard models included the GRACE score as a categorical variable for stroke.

| **Variables** | **HR** | **95% CI** | | **p Value** |
| --- | --- | --- | --- | --- |
|  |  | **Lower** | **Upper** |  |
| The GRACE score | 4.997 | 1.491 | 5.9656 | 0.009 |
| Female | 0.290 | 0.047 | 1.795 | 0.183 |
| Body mass index | 0.907 | 0.743 | 1.107 | 0.335 |
| Ever-smoking | 0.638 | 0.177 | 2.306 | 0.493 |
| Hypertension | 1.167 | 0.286 | 4.764 | 0.829 |
| Hyperlipidemia | 0.637 | 0.130 | 3.122 | 0.578 |
| Diabetes mellitus | 0.217 | 0.040 | 1.177 | 0.076 |
| Stroke history | 2.237 | 0.662 | 7.564 | 0.195 |
| Prior PCI | 4.663 | 1.296 | 16.779 | 0.018 |
| Prior MI | 0.680 | 0.163 | 2.832 | 0.596 |
| Major bleeding history | 3.882 | 0.823 | 18.300 | 0.086 |

Supplementary Table VII Results of multivariable Cox proportional hazard models included the CHA2DS2-VASc score as a continuous variable for all-cause mortality.

| **Variables** | **HR** | **95% CI** | | **p Value** |
| --- | --- | --- | --- | --- |
|  |  | **Lower** | **Upper** |  |
| The CHA2DS2-VASc score | 1.334 | 1.107 | 1.632 | 0.003 |
| Body mass index | 0.906 | 0.821 | 1.000 | 0.051 |
| Ever-smoking | 0.784 | 0.356 | 1.726 | 0.545 |
| Hyperlipidemia | 0.710 | 0.243 | 2.075 | 0.531 |
| Prior PCI | 0.888 | 0.326 | 2.422 | 0.817 |
| Prior MI | 1.113 | 0.450 | 2.749 | 0.817 |
| Major bleeding history | 4.665 | 1.589 | 13.692 | 0.005 |

Supplementary Table VIII Results of multivariable Cox proportional hazard models included the CHA2DS2-VASc score as a categorical variable for all-cause mortality.

| **Variables** | **HR** | **95% CI** | | **p Value** |
| --- | --- | --- | --- | --- |
|  |  | **Lower** | **Upper** |  |
| The CHA2DS2-VASc score | 1.819 | 1.034 | 3.201 | 0.038 |
| Body mass index | 0.904 | 0.818 | 0.998 | 0.045 |
| Ever-smoking | 0.699 | 0.322 | 1.514 | 0.363 |
| Hyperlipidemia | 0.732 | 0.251 | 2.134 | 0.567 |
| Prior PCI | 0.879 | 0.322 | 2.402 | 0.802 |
| Prior MI | 1.169 | 0.474 | 2.883 | 0.735 |
| Major bleeding history | 5.005 | 1.708 | 14.67 | 0.003 |

Supplementary Table IX Results of multivariable Cox proportional hazard models included the GRACE score as a continuous variable for major bleeding.

| **Variables** | **HR** | **95% CI** | | **p Value** |
| --- | --- | --- | --- | --- |
|  |  | **Lower** | **Upper** |  |
| The GRACE score | 1.012 | 1.001 | 1.024 | 0.039 |
| Female | 1.108 | 0.354 | 3.468 | 0.860 |
| Body mass index | 0.998 | 0.939 | 1.060 | 0.940 |
| Ever-smoking | 0.850 | 0.294 | 2.455 | 0.763 |
| Hypertension | 0.910 | 0.316 | 2.617 | 0.860 |
| Hyperlipidemia | 0.370 | 0.132 | 1.041 | 0.060 |
| Diabetes mellitus | 0.811 | 0.305 | 2.157 | 0.675 |
| Stroke history | 0.822 | 0.265 | 2.546 | 0.734 |
| Prior PCI | 0.540 | 0.121 | 2.417 | 0.420 |
| Prior MI | 0.737 | 0.207 | 2.624 | 0.637 |
| Major bleeding history | 1.421 | 0.173 | 11.654 | 0.744 |

Supplementary Table X Results of multivariable Cox proportional hazard models included the GRACE score as a categorical variable for major bleeding.

| **Variables** | **HR** | **95% CI** | | **p Value** |
| --- | --- | --- | --- | --- |
|  |  | **Lower** | **Upper** |  |
| The GRACE score | 2.880 | 1.291 | 6.442 | 0.010 |
| Female | 1.084 | 0.347 | 3.386 | 0.890 |
| Body mass index | 0.997 | 0.940 | 1.057 | 0.911 |
| Ever-smoking | 0.890 | 0.309 | 2.563 | 0.829 |
| Hypertension | 0.980 | 0.342 | 2.812 | 0.970 |
| Hyperlipidemia | 0.396 | 0.142 | 1.111 | 0.078 |
| Diabetes mellitus | 0.782 | 0.294 | 2.078 | 0.621 |
| Stroke history | 0.814 | 0.265 | 2.499 | 0.719 |
| Prior PCI | 0.515 | 0.115 | 2.303 | 0.385 |
| Prior MI | 0.662 | 0.185 | 2.369 | 0.526 |
| Major bleeding history | 1.546 | 0.200 | 11.947 | 0.676 |
